# A rare bladder cancer - small cell carcinoma: review and update

**DOI:** 10.1186/1750-1172-6-75

**Published:** 2011-11-13

**Authors:** Nabil Ismaili

**Affiliations:** 1 Medical oncology, centre régional d'oncologie, Agadir, Morocco

## Abstract

Small cell carcinoma of the bladder (SCCB) is rare, highly aggressive and diagnosed mainly at advanced stages. Hematuria is the main symptom of this malignancy. The origin of the disease is unknown; however the multipotent stem cell theory applies best to this case. Histology and immunohistochemistry shows a tumour which is indistinguishable from small cell lung carcinoma (SCLC). Coexistence of SCCB with other types of carcinoma is common. The staging system used is the TNM-staging of bladder transitional cell carcinoma. The treatment is extrapolated from that of SCLC. However, many patients with SCCB undergo radical resection which is rarely performed in SCLC. Patients with surgically resectable disease (< or = cT1-4aN0M0) should be managed with multimodal therapy associating chemotherapy, surgery and/or radiotherapy. Neoadjuvant chemotherapy using four chemotherapy cycles followed by radical cystectomy is the most effective therapeutic sequence. Patients with unresectable disease (> or = cT4bN+M+) should be managed with palliative chemotherapy based on neuroendocrine type regimens comprising a platinum drug (cisplatin in fit patients). The prognosis of the disease is poor mainly in the case of pure small cell carcinoma. Other research programs are needed to improve the outcome of SCCB.

## Disease name

Small cell carcinoma of the bladder

Poorly differentiated neuroendocrine carcinoma of the bladder

## Definition

Small cell carcinoma of the bladder (SCCB) is a rare, poorly differentiated neuroendocrine epithelial tumour associated with a more aggressive behaviour and poorer outcome than bladder transitional cell carcinoma (TCC). It is mostly diagnosed at advanced stage and generally believed to have a high metastatic potential. Current knowledge of this disease is limited and was based mainly on retrospective investigations. The disease was initially described in 1981 by Cramer et al [1]. Bladder small cell carcinoma (SCC) is frequently found combined with other histological forms of bladder cancer: TCC, adenocarcinoma and squamous cell carcinoma [2-10]. The pathogenesis of primary SCCB is unknown. However, several hypotheses were proposed to explain the origin of SCC in the bladder. The most important hypothesis was: the origin of SCCB may be a multipotential common stem cell. Treatment of SCCB is extrapolated from the treatment of small cell lung carcinoma (SCLC). This comprehensive review would provide a real insight into the epidemiology, pathogenesis, diagnosis, staging, treatment, and prognosis of SCCB.

## Literature review

We based our review on the MEDLINE database using the key words 'bladder cancer', 'small cell carcinoma', 'pathogenesis', 'diagnosis', 'treatment', and 'prognosis'. The research was performed since January 1980 up to July 2011. Only one prospective phase II study was reported in the English literature. Twenty retrospectives studies including ≥ 20 patients have been reported. There have also been several interesting case reports and literature reviews.

## Review

### I - Epidemiology

Small cell cancer of the bladder is an extremely rare bladder malignancy with a mean frequency of 0.7% and a range between 0.35% and 1.8% [2-7]. The reported incidence is less than 1-9/1,000,000 habitant. Since 1980, less than 1000 cases of SCCB have been diagnosed and reported in the literature up to July 2011. The demographic characteristics of SCCB are similar to those seen in patients with transitional cell carcinoma (TCC). The majority of patients are male, with a mean sex ratio equal to 5:1, and a range between 1:1 to 16:1 [2-8,10-15]. Most patients are in the sixth to seventh decade. Mean age at time of first diagnosis is 67 years; ranging between 32 to 91 years [5,8,11,12]. Like TCC, SCCB is often associated with a smoking history (in 65 to 79% of the cases) [4,7-9]. White patients represent the vast majority of cases (74% to 97% of cases) [5,9,12]. Table 1 summarizes the epidemiological and clinical characteristics of SCCB.

**Table 1:** Demographics and clinical characteristics of patients with SCCB

Authors	No	Sex ratio	Age (range)	Smoking history (%)	White race (%)	Symptoms (%)	Frequency of SCC (%)	Percentage of mixed histology
Blomjous 1989[2]	18	2.6:1	69 (50-81)	-	-	Hematuria; Dysuria	0.48%	55.6%

Holmang 1995[3]	25	2.5:1	71.2 (54-87)	-	-	Hematuria	0.7%	60%

Lohrisch 1999[4]	14	1:1	-	79%	-	Hematuria (100%); Local pain (36%)	0.35%	50%

Iczkowski 1999[11]	46	6.7:1	67 (32-91))	-	-	-	-	-

Siefker-Radtke 2004 (MD Anderson)[12]	88	3.3:1	68 (31-87)	-	88%	Hematuria	-	79.5%

Cheng 2004[8]	64	3.3:1	66 (36-35)	65%	-	Hematuria (88%)	-	68%

Mangar 2004[14]	14	6:1	74 (54-91)	-	-	Hematuria (93%)	-	-

Choong 2005[5] (Mayo Clinic)	44	3:1	66.9 (47-88)	-	97.7%	Hematuria (68.2%); Incidental finding (18%); Urinary obstruction (6.8%); Dysuria (2.3%); Abdominal pain (2.3%); Urinary tract infection (2.3%); Ectopic ACTH secretion (2.3%)	0.5%	38.6%

Abrahams 2005[9]	51	4:1	67 (39-87)	-	74%	Haematuria (63%); Dysuria (12%); Abdominal pain (2%); Urinary obstruction (2%); Weight loss (2%); Urinary tract infection (2%)	-	88%

Bex 2005[10]	25	11.5:1	64 (40-90)	-	-	-	-	44%

Quek 2005[6]	25	3:1	68 (40-82)	-	-	-	1%	30%

Mukesh 2008[13]	20	3:1	68	-	-	-	-	-

Ismaili 2008[7]	14	16:1	60.5 (45-78)	78.5%	-	-	1.8%	64.3%

Bex 2009[15]	17	16:1	62 (44-78)	-	-	-	-	50%

Siefker-Radtke 2009 (MD Anderson)[33]	30	14:2	66.2 (43.1-81)	-	-	-	-	43%

Bex 2010[40]	51	4.1:1	65 (57-74)	-	-	-	-	59%

Abbreviations. SCC = small cell carcinoma; No = number of patients

### II - Pathogenesis

Pathogenesis of SCCB is not well defined. However, several hypotheses were proposed to explain the origin of SCC in the bladder. The most important hypotheses were: 1. malignant transformation of bladder neuroendocrine cells gives rise to bladder SCC. This hypothesis was supported by the fact that neuroendocrine cells were found previously in the urinary bladder [16]; 2. SCCB arises from urothelial metaplastic changes [1,17]; and a third and more powerful theory suggests that the origin of SCCB may be a multipotential common stem cell that has the ability to differentiate into various cell types depending on the influence of specific transformation or progression-related gene. This may explain the coexistence of SCCB with TCC, and the heterogeneity of the immunohistochemical staining (cytokeratin and endocrine markers) [18-20].

### III - Clinical features

The clinical features of SCCB are similar to those of bladder TCC and reflect the presence of a tumoral mass. Gross hematuria is the most common symptom in SCCB which was noted in 63 to 88% of the cases [5,8,9,12]. Dysuria has been reported as the second most common symptom [2,9]. Urinary obstruction, abdominal pain, urinary tract infection and weigh loss have been reported occasionally [4,5,9]. Rare cases of paraneoplastic syndromes such as ectopic ACTH secretion and hypercalcaemia were also reported [5,21].

### IV - Diagnosis

Diagnosis of SCCB is mainly accomplished via histopathological examination of specimens obtained by cystoscopy and transurethral resection of the bladder tumour (TURBT) [22]. Immunochemistry staining is extremely helpful in establishing the diagnosis. The role of molecular biology has not yet been defined.

#### (A) Histopathology

**Figure 1:**
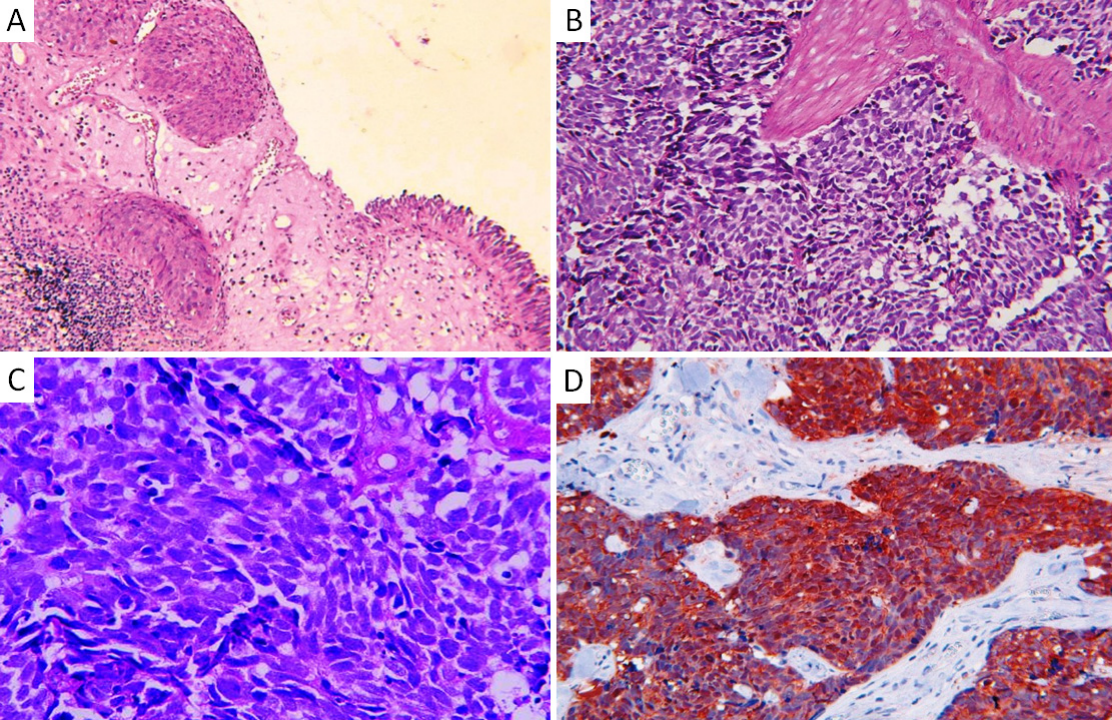
**Pathology of small cell carcinoma of the bladder **[31,43]. A. Hematoxylin and eosin (H and E) staining of the biopsy specimen, low-power view: Urothelial mucosa unfiltered by poorly differentiated carcinomatous proliferation comprised sheets of monomorphic cells. B. Hematoxylin and eosin (H and E) staining of the biopsy specimen, high-power view (×20): Proliferation comprised small cells with hyperchromatic nuclei infiltrating the muscle. C. Hematoxylin and eosin (H and E) staining: High-power view (×40) of a transurethral resection of small cell carcinoma, showing typical scant cytoplasm, increased mitotic index, spindling, and prominent nuclear moulding. D. Immunostaining: NSE-antibody-positive bladder tumor cells

In histological studies, SCCB are identical to SCLC. Therefore, the diagnosis is based on the criteria established by the WHO classification system (2004), used for the diagnosis of SCLC. In light microscopy, morphological studies of SCCB sections stained with haematoxylin and eosin showed packed cells having scant cytoplasm containing few organelles. Tumour is composed of nests of small round malignant cells with pyknotic round to oval nuclei and evenly dispersed "salt and pepper chromatin" (Figure 1A, B and 1C) [9]. The mitotic rate is high (> 10 mitotic figures ⁄ 10 high-power fields) in 57% of the cases. Tumour rosettes were seen in 23.5% of the cases. Tumour necrosis was present in the majority of the cases. Crush artefact (Azzopardi effect) was found in 78.4% of the cases. Vascular invasion was present in 16.7% of the cases [9]. In most reports, the authors showed a higher incidence of mixed SCC [2-10,15]. In Abrahams study, mixtures of SCC with transitional cell carcinoma was present in 70% of the cases, while mixtures of SCC with adenocarcinoma and squamous carcinoma were present only in 8% and 10% of the cases respectively [9].

#### (B) Immunohistochemistry

Immunohistochemistry has a central role for the diagnosis of SCCB through the staining of tumour components by antibody markers targeting the following antigens: neuron-specific enolase (NSE), chromogranin, synaptophysin, serotonin, cytokeratin, S-100 protein, TTF1, EGFR and C-KIT (table 2) [2,9,11,23-28]. The most expressed markers would result on an intense staining of the cytoplasm: NSE (with a frequency of 88.5%) (Figure 1D), synaptophysin (72.4%), and chromogranin (50%) [2,9,11,23]. SCCB are also stained with the epithelial markers: CAM 5.2, CK7, and EMA in 59%, 41%, and 77.7% of the cases, respectively. This supports the urothelial origin of SCCB [2,9,11,24]. TTF-1 expression in SCCB was found in 40% of the tumours in 2 studies, demonstrating that this marker can be expressed in SCC other than those of pulmonary origin [24,25]. Immunochemistry staining of EGFR and C-KIT showed weak cytoplasmic staining in 30% and 27% of the cases, respectively [9,26,27]. PDGFRA expression was reported in one case [28].

**Table 2:** Immunohistochemistry findings in small cell carcinoma of the urinary bladder.

Antibody	No of studies	% of positives staining (mean)
**Neuroendocrine markers**		

NSE[2,9,11,23]	4	25-100% (88.5%)

Synaptophysin[2,11,9]	3	66.6-76% (72.4%)

Serotonin[23]	1	78%

Chromogranin[2,9,11,17]	4	22-89% (50%)

**Epithelial markers**		

Cytokeratin[2,23]	2	70-77% (75%)

EMA[2]	1	77.7%

CK7[24]	1	59%

CAM 5.2[2,11,9]	3	47-66.6% (41%)

**Other markers**		

S-100 protein[23]	1	40%

TTF1[24,25]	2	39-50% (40%)

EGFR[9,26]	2	27-36% (28.6%)

C-KIT[9,27]	2	22-27% (27%)

CD44v6[11]	1	7%

PDGFR[28]	1 case report	+

Abbreviations. NSE = neuron specific enolase; EMA = epithelial membrane antigen; CK7 = cytokeratine 7; EGFR = epidermal growth factor receptor; PDGFR = platelet derived growth factor

#### (C) Molecular genetics

Genetic alterations in SCCB have been the subject of few studies, because of the rarity of the disease. A Comparative genomic hybridization (CGH) study has demonstrated chromosomal deletions at 10q, 4q, 5q and 13q [18,29]. These regions are frequently deleted in human tumours and known to carry some tumour suppressor genes: PTEN located at 10q23 and the retinoblastoma gene located at 13q14 [30]. Additions of DNA sequences have been reported at 5p, 6p, 8q and 20q [18,29]. However, no clear single genetic lesion has been characterized. Other studies are necessary to define the role of molecular genetics in the diagnosis of SCCB.

### V - Bladder small cell cancer imaging

As for TCC of the bladder, the most widely used imaging examination of SCCB is the pelvic computed tomography scan of the bladder mass and the locoregional extension (bladder wall and pelvic lymph nodes).

### VI - Staging

**Figure 2:**
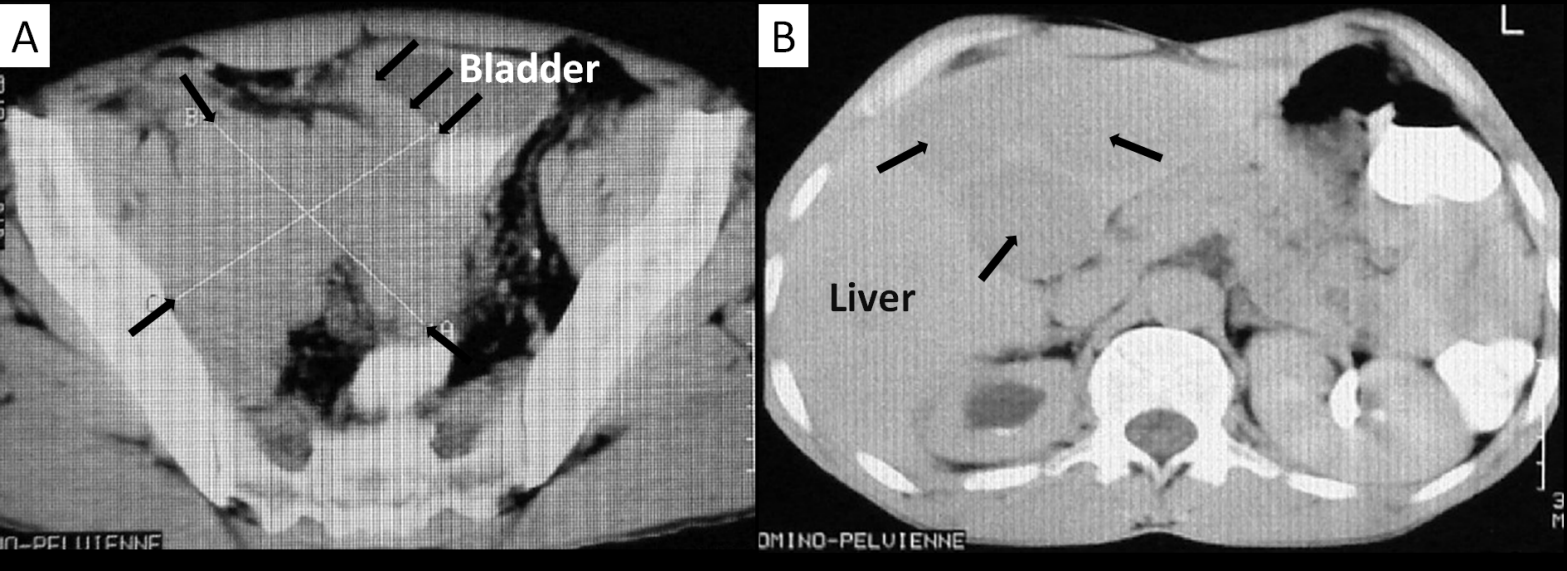
**Bladder small cell carcinoma imaging **[31]. A. Computed tomography scan of the pelvis shows a heavy tumor at the right bladder wall with intraluminal and extravesical extension (arrows). B. Computed tomography scan of the abdomen shows a multinodular liver disease from bladder small cell carcinoma (arrows).

In most cases, the diagnosis is made at advanced stages (T3-T4/N+/M+) (Figure 2A) [31]. More than 95% of SCCB cases are diagnosed at muscle invasive stage T2 or more [5-9,11,12]. As an example, in a large MD Anderson series of 88 cases, only 4.5% (4 patients) were diagnosed at superficial stage of the disease (Ta/T1), while 40.1% (n = 36) were diagnosed at stage T2, 28.3% (n = 25) were diagnosed at stage T3-T4a (stage III) and 26.1% (n = 23) were diagnosed at stage T4b-M+ (stage IV) [12]. Similar findings were observed in three others larges series [5,8,11]. As for bladder TCC, the TNM-staging system was commonly used for SCCB [2,3,5-8,14,12,32,33]. Patients with SCCB restricted to the bladder, should be considered as having surgically resectable disease (≤T1-4aN0M0) [33]. In this case, treatment with neoadjuvant chemotheapy followed by surgery is favored. Patients with regional or non regional lymph nodes (retroperitoneal lymph nodes or distant lymph nodes) or with distant metastasis have the disease at advanced stage (surgically unresectable disease) (≥cT4bN+M+) [33]. Systemic chemotherapy is the treatment of choice for these patients.

Based on two large studies, the most frequent sites of metastasis were pelvic and retroperitoneal lymph nodes (28.6% - 53%), liver (23.8% - 47%) (Figure 2B), bone (23.8 - 33%), brain (7.9% - 16%) and lung (9.5% - 13%) [5,12]. Consequently, the staging of SCCB should include computed tomography scan of the pelvis, abdomen chest, brain, and bone scan.

### VII - Differential diagnosis

SCCB must be differentiated from several other cancers [23]:

*Direct invasion of the bladder by SCC of the prostate; prostatic small cell carcinoma is typically negative for prostate-specific antigen.

*Metastatic SCC from another source, usually from the lung. Metastatic SCLC may not be distinguishable histologically from a primary SCCB; however, the presence of TCC component (including TCC in situ) would support a diagnosis of bladder SCC.

*Primary lymphomas of the bladder; lymphomas are positive for leukocyte common antigen (LCA), and negative for keratin and neuroendocrine markers.

### VIII - Disease management

Because of the rarity of SCCB, there is no standard treatment of the disease. SCCB is an aggressive tumour (90% of patients are at stage II or more and 25% are at stage IV). This favours the use of chemotherapy (CT) in the management of the disease [12]. Table 3 summarizes the most important studies addressing the management of SCCB.

**Table 3:** Treatment strategies and outcome of bladder small cell carcinoma according to the most important studies published in the English literature.

Authors	No	Study design	Stages (No)	Treatments (No)		Results and comments
Blomjous 1989 [2]	18	Retrospective	T2(5) T3(8) T4(5)	CT group	TURBT→RT→CT(2) TURBT→CT(1) RC→CT(1) CT→RC(1)	-OS and 2 years survival in the whole group = 9 months and 27.7% respectively -OS and Survival at 2 years in CT group vs. no CT group = NR vs. 7 months and 60% vs. 15.5%, respectively
					
				Non-CT group	TURBT→RT(9) RC(3) None(1)	

Holmang 1995 [3]	25	Retrospective	T2(7) T3(10) T4(2) IVM+(6)	RC→RT(18) CT(2) None(5)		5 years survival in the whole group = 20%

Lohrisch 1999 [4]	14	Retrospective	LD(9) ED(1)	CT group	CT→RT(8) CT→RC(1) CT→C(1)	-OS in the CT grope = 41 months -Survival at 5 years = 70% in the CT group vs. 0% in the non CT group
				
			LD(2); ED(2)	Non-CT group	RT(2) None(2)	

Bastus 1999 [32]	5	Retrospective	T2(1) T3(3) T3N1(1)	CT→RT(5)		-All patients were treated with sequential chemo-radiotherapy; -2 years survival in the whole group = 80%

Siefker-Radtke 2004 (MD Anderson) [12]	46	Retrospective cohort	T2(13) T3-T4a(8)	CT→RC(21)		5 years survival in neoadjuvant CT group was significantly better than surgery alone group = 78% vs. 36%, *p *= 0.026
				
			T2(12) T3-T4a(7) Unknown(n = 6)	RC(25)		

Cheng 2004 [8]	64	Retrospective cohort	T1(1) T2(30) T3(29) T4(4)	RC(38) RT(10) CT(23)		No difference in survival between RC group vs. non-RC group

Mangar 2004 [14]	14	Retrospective	T3(8) T3N1(1) T4 (2) IVM+(3)	RC group	RC→CT→RT(1) RC→RT(3) RC(2)	Outcome in RC group > outcome in non-RC group
					
				Non-RC group	PRT(5) None(3)	

Choong 2005 (Mayo Clinic) [5]	44	Retrospective	II(12)	RC(7) NCT→RC(1) PC(3)		-5 years survival in the whole group = 25% -5 years survival in stage II > III/IV = 63%, 15%, and 10% respectively, *p*< 0.001; -No difference between stages III and IV
				
			III(13)	RC(8) RC→CT(2)		
				
			IV(19)	RC→CT(10) RC(2) CT(5)		

Bex 2005 [10]	25	Prospective	LD(10) ED(3)	CT group	CT(13)→RT(8)	CT > non-CT (OS = 15 vs. 4 months respectively, *p *= 0.003)
				
			LD(7) ED(5)	Non-CT group	RT(5) RC(3) P(4)	

Quek 2005 [6]	25	Retrospective	I/II(4) III(2) IV N+ or M+(19)	RC→ACT(13) NCT→RC(1) RC(11)		-Survival in mixed SCCB > survival in pure SCCB, *p *= 0.06 -RC + ACT > RC alone

Mukesh 2008 [13]	20	Retrospective	LD(11); ED(9)	CT group(13)	CT→RT(6) RC→CT(7)	Outcome in CT group > outcome in non-CT (OS = 33 months vs. 3 months, respectively)
					
				Non-CT group(7)	BSC(4) RC(4) RT(1)	

Ismaili 2008 [7]	14	Retrospective	II(4) III(5) IVM0(5)	RC→CT(4) RC(5) CT→RC(2) CT(1) RCT(1) None(1)		-Survival in mixed SCCB > survival in pure SCCB, *p *= 0.01, -CT + Surgery > Surgery

Bex 2009 [15]	17	Retrospective	LD(17): -T2(14) -T3(2) -T4a(1)	CT→RT (60: 56-70Gy) (17) Salvage RC (3)		-All patients have been treated with sequential chemoradiotherapy -OS = 32.5 months -2, 3, and 5 years survival = 56%, 47%, and 36% respectively

Siefker-Radtke 2009 (MD Anderson) [33]	30	Phase II	Resecable patients(18): T2N0M0	CT→RC		-5 years survival in operable group = 80% -OS = 58 months vs 13.3 months, in operable vs non operable patients, respectively -Incidence of brain metastasis in stage III/IV = 50%
				
			Unresecable patients(12): T3b-4aN0M0	CT alone		

Bex 2010 [40]	51	Retrospective	LD(39)	CT→RT		-Survival of patients with LD = 35 months vs 6 months in patients with ED. -Incidence of brain metastasis = 10.5%
				
			ED(12)	CT		

Abbreviations. OS = overall survival; NS = no significant; RC = radical cystectomy; TURBT = transurethral resection of the bladder tumour; ACT = adjuvant chemotherapy; NCT = neoadjuvant chemotherapy; PC = partial cystectomy; CT = chemotherapy; RCT = concurrent chemoradiotherapy; PRT = palliative radiotherapy; NR = no reached; LD = limited disease; ED = extensive disease; SCCB = small cell carcinoma of the bladder; Definition for LD (limited disease): in analogy to SCLC, patient with any local stage, no distant metastases and involvement of maximally one loco regional lymph node less than 2 cm in imaging (cTx cN0-1 M0) [15]; Definition for ED (extensive disease): unresectable and metastatic disease [15].

#### (A)Radical resection

In contrast with SCLC, more than half of the patients with SCCB undergo *radical resection *[3,5-8,12]. In a review of 88 cases, reported by MD Anderson Cancer Centre, 46 patients undergone cystecomy [12]. Similarly in two other studies, the radical resection was performed in 60 to 70% of the cases [5,8]. Surgery was favoured because of the frequent combination of SCC with TCC. In fact, in one study, 60% of the patients having SCCB developed TCC, 24 to 26 months after the completion of curative chemo-radiotherapy (CRT) [4]. However, in a multi-institutional review of 64 patients with localised SCCB, the efficacy of cystectomy has been questioned as no survival difference was found between patients undergoing surgery and those without surgery (5-year survival was 16% vs. 18%, respectively) [8]. Surgery alone is not appropriate to achieve cure for patients with SCCB. In the retrospective study conducted by MD Anderson, the patients who received neoadjuvant CT have significantly better survival than those who did not receive neoadjuvant CT [12].

#### (B)Radiotherapy

In general, SCLC is treated with a combination of radiotherapy (RT) and CT. In analogy to SCLC, RT either alone or in combination with CT, was used to treat SCCB at localised disease [3,4,10,15,32].

Three retrospectives studies with longer follow-up (5 years), have assessed the role of curative RT in the management of localised bladder SCC [3,4,15]. In the first study (n = 25), a group of 18 patients received surgery and curative radiotherapy (without chemotherapy) [3]. In the 2 others studies, 10 and 17 patients, respectively, received sequential chemo-radiotherapy [3,4]. The 5 years survival was equal to 28%, in the first study, vs. 70% and 36% in the second and third studies, respectively [3,4,15]. Long-term survivors have been reported (up to 18 years) [3], however, those with longer follow-up suggest a higher likelihood of relapse over time [4]. These results confirmed that radiotherapy can be curative, but significantly more curative when used in combination with chemotherapy.

#### (C) Chemotherapy

Chemotherapy is the major treatment modality for SCCB [34,35]. In one large series, the authors showed on multivariate analysis that cisplatin chemotherapy is the only predictor factor for survival of SCCB patients (p < 0.0001) [35]. In surgically resectable disease chemotherapy is used as neoadjuvant therapy to shrink the tumour prior to local therapy or as adjuvant treatment after surgical resection [5,12].

##### Neoadjuvant chemotherapy

Neoadjuvant CT before surgery in surgically resectable SCCB has been investigated in several retrospective studies and in one phase II prospective study [12,33]. In addition primary CT was used in sequence with radiation to increase the efficacy of RT [4,10,15,32].

Neoadjuvant CT in bladder SCC cancer has four theoretical advantages [36,37]:

*the early treatment of micrometastatic disease,

*the systemic treatment is better tolerated by allowing the preoperative administration of CT drugs in optimal doses with less toxicity,

*SCCB is highly chemosensitive disease; the vast majority of patients have great responses,

*downstaging, which facilitates the surgical techniques.

One retrospective cohort study and one phase II clinical trial demonstrated the advantage of CT in neoadjuvant setting.

In the MD Anderson retrospective study, 46 operable patients were included; the first group of patient (n = 21) was treated with 4 cycles of neoadjuvant sequential CT regimen based on ifosfamide plus doxorubicin at day 1 repeated every 42 days and etoposide plus cisplatin at day 21 repeated every 42 days; the second group was treated with surgery alone (n = 25). At last follow-up, 5-year survival was significantly higher in CT group: 78% versus 36% in surgery alone group (*p *= 0.026) [12]. In addition, the results of the MD Anderson phase II clinical trial recently published, confirmed theses results. In this prospective study, 30 eligible patients were included, eighteen of them were surgically resectable and 12 were surgically unresectable. Operable patients have been treated with neoadjuvant CT followed by surgery. At last follow-up, OS and 5 years survival in resectable group was equal to 58 months and 80%, respectively [33].

Based on these data, neoadjuvant CT should be considered as the treatment of choice of surgically resectable SCCB.

##### Adjuvant chemotherapy

No clear data defines the role of adjuvant CT after primary surgery of invasive bladder SCC. Only one retrospective study conducted by the University of Southern California has addressed this question. In the published article, the authors concluded that adjuvant CT may provide improved survival compared with cystectomy alone [6]. In addition, the Mayo Clinic recommendations propose cystectomy alone for patients with stage II disease, and adjuvant chemotherapy for patients with stage III and VI (M0) disease [5]. However, it is important to note that many institutions who followed the Mayo recommendations of initial cystectomy report very poor outcomes and high likelihood of upstaging [5,6].

##### Chemotherapy in advanced disease

When SCCB arise outside the bladder, CT plays a prominent role in the management of these tumors. In metastatic setting, the most commonly used regimen for SCCB is cisplatin plus etoposide CT in analogy to SCLC [5,12,15]. Etoposide is administered at 100 to 120 mg/m^2 ^intravenously on day 1 to 3, repeated every 3 weeks. Cisplatin is usually given at 70 to 100 mg/m^2 ^intravenously on day 1. The MD Anderson group showed that preoperative CT with a neuroendocrine regimen was more likely to successfully eradicate the small cell component compared to regimens typically used for TCC. In fact, of the 12 patients treated with a neuroendocrine regimen only 2 had small cell carcinoma present at cystectomy. However, for those 9 patients treated with a transitional cell carcinoma regimen (MVAC) 6 had small cell carcinoma still present at cystectomy [12]. Consequently, this group recommended the protocols used in the neuroendocrine tumours containing etoposide and cisplatin or ifosfamide and doxorubicin for both histological types: pure SCC and mixed SCC [38]. Other authors recommended a regimen covering both small cell component and TCC component for mixed SCCB: the addition of taxane or ifosfamide to the standard platinum plus etoposide regimen may be considered [39]. In the unfit patient, cisplatin should be substituted with carboplatin.

Other chemotherapy regimens including etoposide-cisplatine alternating protocol either with ifosfamide-doxorubicin or with cyclophosphamide, doxorubicin and vincristin (CAV), as well as single agents, including paclitaxel, irinotecan, topotecan, and doxorubicin, have all been used in SCCB [5,12]. Table 4 summarizes the most used regimen in the management of SCCB.

**Table 4:** Chemotherapy regimens used in the treatment of SCCB

Regimen	Schedule	Drugs and doses				
First line						

EP (IV)[5,10,15,33]	On day 1 to 3, repeated after 21 days	Etoposide 120 mg/m^2 ^on day 1 to 3	Cisplatin 80-100 mg/m^2 ^, on day 1			

IA/EP (IV)[12,33]	Alternative regimen: ifosfamide plus doxorubicin on day 1 to 3 repeated every 42 days and etoposide plus cisplatin on day 22 to 26 repeated after 42 days	Ifosfamide 2 g/m^2 ^, on day 1 to 3	Doxorubicin 25 mg/m^2 ^, on day 1 to 3	Etoposide 80 mg/m^2 ^, on day 22 to 26	Cisplatin 20 mg/m^2 ^, on day 22 to 26	

VIP (IV)[10]	On day 1 to 4, repeated after 21 days	Ifosfamide 1.2 g/m^2 ^, on day 1 to 4	Etoposide 75 mg/m^2 ^on day 1 to 4	Cisplatin 20 mg/m^2 ^on day 1 to 4		

EP/CAV (IV)[11]	Alternative regimen: EP on day 1 to 3 repeated after 42 days and CAV on day 22 repeated every 42 days	Etoposide 100 mg/m^2 ^on day 1 to 3	Cisplatin 80 mg/m^2 ^, on day 1	Cyclophosphamide 800 mg/m^2 ^on day 22	Doxorubicine 50 mg/m^2 ^on day 22	Vincristine 1.4 mg/m^2 ^on day 22

MVAC (IV)[12]	On day 1, 2, 15, and 22, repeated every 28 days	Methotrexate 30 mg/m^2 ^on day 1, 15 and 22	Vinblastine 3 mg/m^2 ^on day 2, 15, and 22	Doxorubicin 30 mg/m^2 ^on day 2	Cispatin 70 mg/m^2 ^on day 2	

**Second line**						

Topotecan (IV)[5]	On day 1 to 5, repeated every 21 days	Topotecan 1.5 mg/m^2 ^on day 1 to 5				

CAV (IV)	On day 1, repeated every 21 days	Cyclophosphamide 800 mg/m^2 ^on day 1	Doxorubicin 50 mg/m^2 ^on day 1	Vincristine 1.4 mg/m^2 ^on day 1		

Vinorelbine (IV)[41]	On day 1, 8, and 15. The cycle is repeated every 21 days	Vinorelbine 25 mg/m^2 ^on day 1, 8, and 15				

IV = intravenous

#### (D) Nervous system and bone metastasis

Based on the high efficacy of chemotherapy against metastatic small cell carcinoma, palliative radiotherapy is rarely adopted. However, radiotherapy is reserved for treatment of symptomatic brain metastases, symptomatic bone metastases and cord compression. According to a recent retrospective investigation, the incidence of symptomatic brain metastases from SCCB is significantly lower than that from SCLC. Therefore, the authors do not recommend systematic prophylactic brain irradiation (PCI) in patients with SCCB [40]. In another hand, the authors at MD Anderson, report in the phase II clinical trial a 50% incidence of brain metastases in patients with stage III-IV disease; this information suggests a possible group to consider for PCI [33].

#### (E) Progressive or relapsing disease

In analogy to SCLC, the likelihood of response to further CT can be predicted on the basis of the response to previous therapy and the duration of free interval. Patients who did not respond to previous therapy or who relapsed within 3 months are judged refractory. For patients with sensitive disease, the same induction regimen can be used for treatment. Three weekly vinorelbine has been tested in a case series and has showed an interesting activity [41]. Second-line regimens are summarized in table 4.

#### (F) Future directions

Despite the promising results obtained by chemotherapy based on cisplatin, the majority of patients die of metastatic disease.

**Figure 3:**
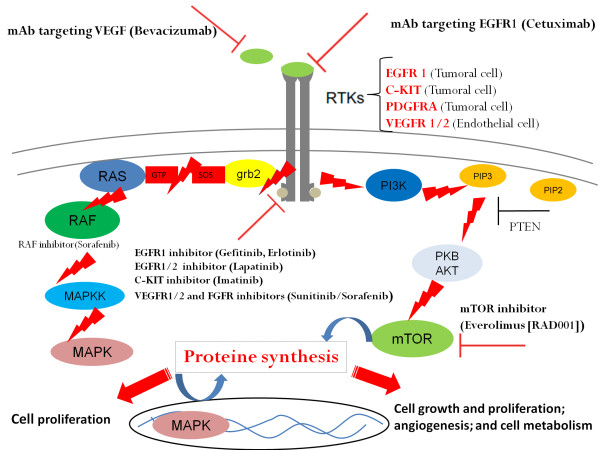
**Deregulated signaling pathways and targeted therapy which should be evaluated in the future in SCCB in analogy to SCLC**. **Abbreviations: **EGFR, Epidermal Growth Factor Receptor; VEGFR, Vascular Endothelial Growth Factor R; FGFR: Fibroblast Growth Factor Receptor; PDGFR, Platelet Derived Growth Factor Receptor; mTOR: mammalian Target of Rapamycin.

The progress in molecular biology has led to the investigation of new molecules in several primary tumours including SCLC. Overexpression of several receptors such as the VEGFR (vascular endothelial growth factor receptor) on endothelial cells, the EGFR (epidermal growth factor receptor, the c-KIT, the PDGFR (platelet derived growth factor receptor) and the FGFR (fibroblast growth factor receptor), on tumor cells has prompted the scientific community to evaluate the efficacy and safety of new molecules targeting signaling pathways controlled by these proteins in metastatic SCLC (bevacizumab, sunitinib, sorafenib, pazopanib, Imatinib, cetuximab, erlotinib, Gefitinib, lapatinib, everolimus, bortezomib) (Figure 3). According to preliminary studies, targeting angiogenesis would be the most promising strategy [42]. In analogy to SCLC, the role of theses molecules in metastatic SCCB should be defined in the future.

### IX-Treatment recommendations [39,43-45]

#### (A) Surgically resectable disease

Neoadjuvant chemotherapy followed by radical resection should be considered as the treatment of choice in surgically resectable SCCB. This sequence can achieve a cure in 78-80% of the patients [12,33];

Sequential chemo-radiotherapy is a second treatment option which can achieve a cure in 36 to 70% of the cases [4,15];

In the case when surgery was performed first, adjuvant chemotherapy or adjuvant chemo-radiotherapy should be indicated [5,6];

#### (B) Advanced disease

In advanced stages, chemotherapy based on cisplatin should be considered as the treatment of choice for patients with good performance status (0-1) and good renal function-Glomerular filtration rate (GFR) > 60 mL/min. The treatment should be based on neuroendocrine regimens type etoposide plus cisplatin or the sequential protocol; ifosfamide plus doxorubicin at day 1 and etoposide plus cisplatin at day 21 (table 4). In unfit patients, cisplatin should be substituted by carboplatin AUC 5 to 6.

#### X-Prognosis

The prognosis of SCCB is poor. Five-year survival rate of all stages combined is equal to 19% (16 to 25%) [5,8]. Based on one large study, the 5-year survival rates for patients with Stage II, III, and IV were 63.6%, 15.4%, and 10.5% respectively. Advanced stages III and IV have poorer outcome than stage II disease; *P <*0.0001 [5]. In addition, according to 2 series, pure small cell histology was shown to have poorer outcome than the mixed small cell histology [6,7,34]. Because of the rarity of this disease, no others prognostic factors were identified.

#### XI-Conclusions

Primary SCCB is a rare and aggressive tumour. In more than 50% of the cases, the diagnosis is performed at advanced stages III/IV. Demographic and clinical features are comparable to those of bladder TCC. The origin of disease is not clearly defined; but the multipotent theory is the most accepted. Criteria of pathological diagnosis and radiological work-up are similar to those of SCLC. Coexistence of SCCB with other types of carcinoma is common. Immunochemistry plays a major role in the diagnosis using the markers of neuroendocrine tumours. The staging system mostly used is the TNM-staging system of bladder TCC. The best treatment for this tumour was not established for certain; only one prospective study was published up to now. The strategy of therapy was extrapolated from SCLC. In surgically resectable disease, the management should include multimodal therapy with chemotherapy first followed by radical resection or radical radiotherapy. In advanced disease, chemotherapy using platinum agent (cisplatin in fit patients) is the mainstay treatment. The prognosis of SCCB is poor. Pure small cell histology shows to have worsened prognosis than the mixed small cell histology. Further investigations are needed to improve our knowledge in the diagnosis and treatment of this rare disease.

## Abbreviations

AD: advanced disease; CAM 5.2: cytokeratin marker; CAV: cyclophosphamide, doxorubicin and vincristin; CGH: comparative genomic hybridization; CK7: cytokeratin 7; CT: chemotherapy; EGFR: epidermal growth factor receptor; C-KIT: Proto-Oncogene encoding a transmembrane protein-kinase; EMA: epithelial membrane antigen; LD: localised disease; MVAC: methotrexat, vimblastin, doxorubicin and cisplatin; PTEN: Phosphatase and Tensin Homolog; RT: radiotherapy; SCC: small cell carcinoma; SCCB: small cell carcinoma of the bladder; SCLC: small cell lung cancer; TCC: transitional cell carcinoma; TTF1: thyroid transcription factor 1; TURBT: transurethral resection of the bladder tumour.

## Competing interests

The author declares that they have no competing interests.

## Authors' contributions

NI is involved in concept design, in data collection, drafting and critically revising the manuscript.
